# Preliminary findings on diagnostic performance of computed tomography perfusion images for intracranial arterial stenosis: a retrospective study

**DOI:** 10.1186/s12883-024-03554-x

**Published:** 2024-02-09

**Authors:** Hui Liu, Dan Wu, Zhi-bin Chen, Qian Xiao, Ji-wei Cheng, Xiao-yan Xie, Dong-xiao Qu, Jie Tao, Wei-zhong Wang, Yi-feng Peng, Guo-yi Li, Ying-feng Weng

**Affiliations:** 1 Department of Neurology, Putuo Hospital, Shanghai University of Traditional Chinese Medicine, Shanghai, 200062 P.R. China; 2 Department of Radiology, Putuo Hospital, Shanghai University of Traditional Chinese Medicine, Shanghai, 200062 P.R. China; 3 Central Laboratory, Putuo Hospital, Shanghai University of Traditional Chinese Medicine, Shanghai, 200062 P.R. China

**Keywords:** Computed tomographic perfusion, Computed tomographic angiography, Artery, Stenosis

## Abstract

**Objectives:**

Computed tomographic perfusion (CTP) can play an auxiliary role in the selection of patients with acute ischemic stroke for endovascular treatment. However, data on CTP in non-stroke patients with intracranial arterial stenosis are scarce. We aimed to investigate images in patients with asymptomatic intracranial arterial stenosis to determine the detection accuracy and interpretation time of large/medium-artery stenosis or occlusion when combining computed tomographic angiography (CTA) and CTP images.

**Methods:**

We retrospectively reviewed 39 patients with asymptomatic intracranial arterial stenosis from our hospital database from January 2021 to August 2023 who underwent head CTP, head CTA, and digital subtraction angiography (DSA). Head CTA images were generated from the CTP data, and the diagnostic performance for each artery was assessed. Two readers independently interpreted the CTA images before and after CTP, and the results were analyzed.

**Results:**

After adding CTP maps, the accuracy (area under the curve) of diagnosing internal carotid artery (R1: 0.847 vs. 0.907, R2: 0.776 vs. 0.887), middle cerebral artery (R1: 0.934 vs. 0.933, R2: 0.927 vs. 0.981), anterior cerebral artery (R1: 0.625 vs. 0.750, R2: 0.609 vs. 0.750), vertebral artery (R1: 0.743 vs. 0.764, R2: 0.748 vs. 0.846), and posterior cerebral artery (R1: 0.390 vs. 0.575, R2: 0.390 vs. 0.585) occlusions increased for both readers (*p* < 0.05). Mean interpretation time (R1: 72.4 ± 6.1 s vs. 67.7 ± 6.4 s, R2: 77.7 ± 3.8 s vs. 72.6 ± 4.7 s) decreased when using a combination of both images both readers (*p* < 0.001).

**Conclusions:**

The addition of CTP images improved the accuracy of interpreting CTA images and reduced the interpretation time in asymptomatic intracranial arterial stenosis. These findings support the use of CTP imaging in patients with asymptomatic intracranial arterial stenosis.

## Introduction

According to TOAST, the most common stroke etiology was small vessel occlusion, followed by large artery intracranial disease (posterior cerebral arteries, basilar and vertebral arteries), cardioembolic, and cryptogenic [1, 2]. Aging, systolic hypertension, diabetes, high LDL cholesterol levels and metabolic syndrome are the main risk factors [3]. Transcranial Doppler and magnetic resonance angiography are the most commonly used auxiliary tests during screening and follow-up. CTA can be used as a screening tool to detect intracranial artery stenosis (IAS) and, increasingly, as a confirmatory test to approximate the diagnostic accuracy of ductal DSA, which is still considered the gold standard [4]. There is increasing evidence supporting the role of computed tomographic perfusion (CTP) in acute ischemic stroke (AIS) [5, 6]. CTP is typically performed along with computed tomographic angiography (CTA) to select patients for endovascular treatment in the late time window (6–24 h from symptom onset or last-seen-well). Recent studies have demonstrated that the combination of CTP and CTA can diagnose intracranial large- and medium-artery occlusions more accurately than CTA alone in ischemic stroke [7]. This combination also reduces the reader interpretation time [8]. However, the value of CTP imaging in patients with asymptomatic intracranial arterial stenosis has not yet been sufficiently studied.

Patients with asymptomatic intracranial arterial stenosis require more clinical attention because they may experience worse outcomes in the future than those with normal large intracranial arteries. We aimed to determine the accuracy for readers and interpretation time among patients with asymptomatic intracranial arterial stenosis once the perfusion distribution is considered when combining CTA and CTP images.

## Materials and methods

### Data selection

This retrospective study was approved by the Ethics Review Board of Putuo Hospital Affiliated to Shanghai University of Traditional Chinese Medicine: Shanghai PuTuo District Center Hospital (PTEC-A-2020-66-1; Shanghai, China), all methods were performed in accordance with the relevant guidelines and regulation and the need for informed consent was waived. A total of 47 patients were enrolled, and we recorded simultaneous CTP, CTA, and digital subtraction angiography (DSA) data. The CTA images were derived from the CTP data, and there was no CTP data with motion artifacts.

Eight patients had no history of cerebral infarctions. Among them, four individuals had a milder subarachnoid hemorrhage (two cases of ruptured intracranial aneurysm, one case with no detected intracranial aneurysm, and one individual with an arteriovenous fistula), while three individuals had intracranial arterial stenosis or occlusion. There were 39 patients with cerebral infarctions, including acute or previous infarctions.

**Fig. 1:**
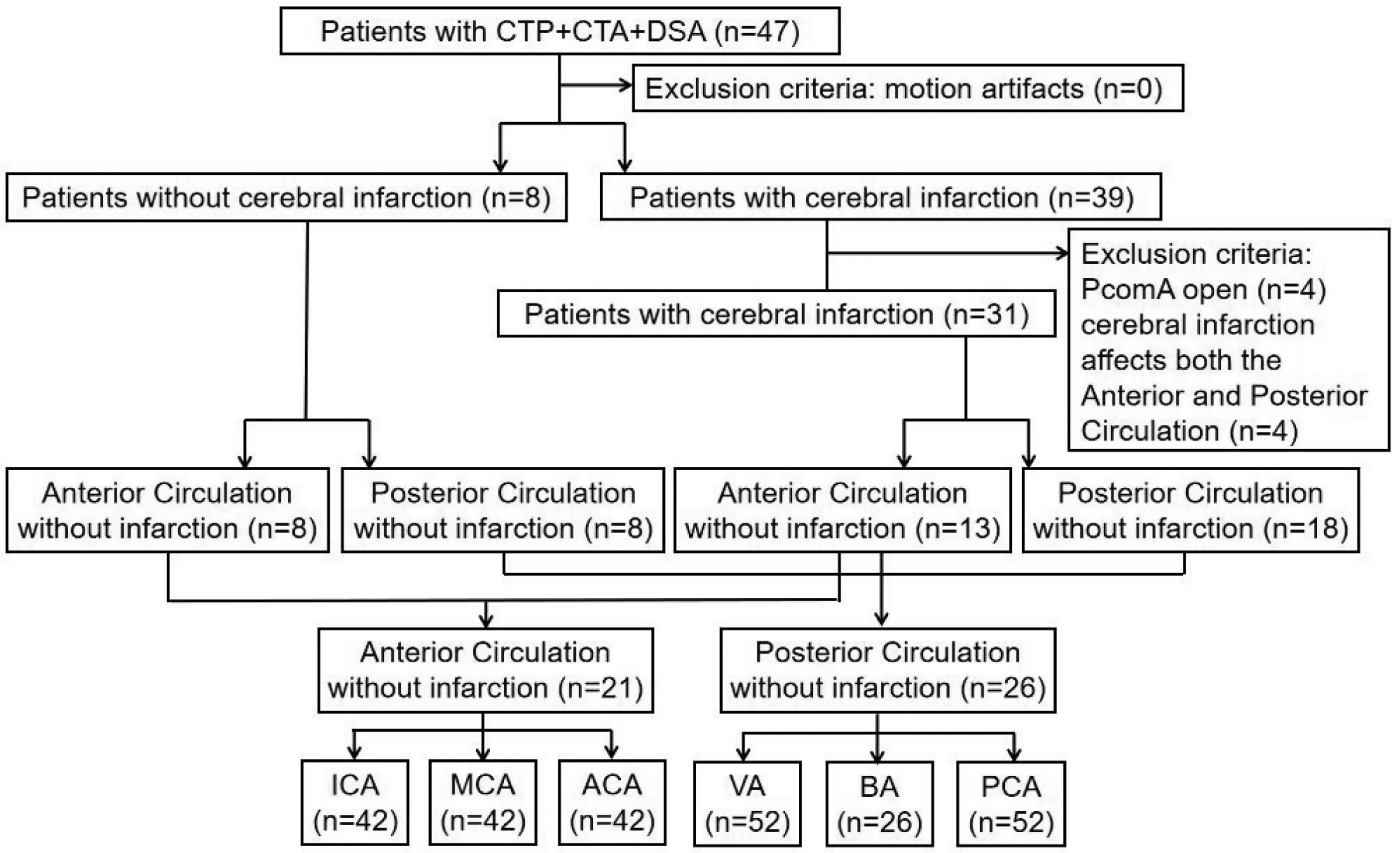
Flowchart showing patient enrollment and exclusion. CTP, computed tomography perfusion; CTA, computed tomography angiography; DSA, digital subtraction angiography; PcomA, posterior communicating artery; ICA, internal carotid artery; MCA, middle cerebral artery; ACA, anterior cerebral artery; VA, vertebral artery; BA, basilar artery; PCA, posterior cerebral artery

We focused on patients with asymptomatic intracranial arterial stenosis. To obtain a more diverse sample, we collected data separately for the anterior and posterior circulations for asymptomatic intracranial arterial stenosis patients (confirmed by head MRI or, if no MRI data were available, by excluding stroke diagnosis based on head CT, clinical symptoms and examination, medical history, and follow-up history). For patients with anterior circulation infarctions, we collected data on asymptomatic intracranial arterial stenosis of the posterior circulation. For patients with posterior-circulation infarctions, we collected data on asymptomatic intracranial arterial stenosis of the anterior circulation. If the DSA indicated a posterior communicating artery (PcomA) open to the patient, their data were excluded. Ultimately, we obtained 21 sample groups for anterior circulation and 26 for posterior circulation. We finally included 42 internal carotid artery (ICA) samples, 42 middle cerebral artery (MCA) samples, 42 anterior cerebral artery (ACA) samples, 52 vertebral artery (VA) samples, 26 basilar artery (BA) samples, and 52 posterior cerebral artery (PCA) samples. Figure 1 shows a flowchart of patient enrollment and exclusion.

### Image acquisition

All CTP and CTA data were acquired using the same CT scanner (192-slice, SOMATOM Force; Siemens AG, Forchheim, Germany). Each patient received an injection of 50 ml of contrast agent at a rate of 5 ml/s, followed by an injection of 20 ml of saline at the same rate. The first CTP frame was obtained 3 s after the start of contrast agent injection. During the CTP examination, 25 sets of images were collected for each patient. The frame rate for the first 19 sets was 1.5 s, and that for the remaining six sets was 3 s. All data were obtained using low-dose radiation with the following scanning parameters: 70 kV, 200 mAs, and a radiation dose (CT Dose Index; CTD Ivol) of 166 mGy. CTP maps were generated using commercial software (Syngo.via CT Brain Perfusion VB30, Siemens Healthcare), which utilizes a deconvolution model with a delay-insensitive algorithm and interhemispheric comparison. A cerebral blood flow (CBF) of < 30% was defined as non-viable tissue, and a Tmax > 6 s was defined as low perfusion.

The CTA images were derived from CTP data. Conventional 3D projections of CTA are routinely performed in our hospital, whereas 3D projections of CTP images are not.

### Data measurement

Two readers who were blinded to the final radiology report, DSA, MRI report, and clinical presentation independently interpreted only the CTA images (*n* = 39). One of the readers was an attending physician working in the Neurology Department (R1), and the other was a radiologist working in the Radiology Department (R2). R1 has over 10 years of clinical practice experience who diagnoses and treats around 240–300 cases of cerebrovascular disease patients per month. R2 has over 17 years of clinical practice experience, and his daily work involves more than 20 cases of brain CTA scans and around 5 cases of brain CTP scans. The presence/absence and degree of stenosis, affected arteries, and side were all recorded by an independent observer who was not involved in image interpretation. The observer also recorded interpretation times for both readers.

To avoid recall bias, the sample order was rearranged after one week reading CTA images alone, the same two readers analyzed the CTA and CTP images (using cerebral blood volume (CBV), CBF, TTP, and Tmax maps) independently, and the observer recorded the results and interpretation time again. The researcher collected data for the left and right hemispheres separately when summarizing the data. The classification of the vasculature for statistical analysis was as follows: anterior circulation included the ICA, MCA, and ACA; whereas posterior circulation included the VA, BA, and PCA. The affected vascular sites were not further subdivided. The readers did not estimate the percentage of vascular stenosis during image interpretation; instead, they classified the degree of stenosis into five categories: normal, mild, moderate, severe, and occluded. The degree of the lesion in each vessel was based on the narrowest segment. Anomalies of the ACA, such as the bilateral common trunk or absence of one side of the ACA, were documented as normal. Embryonic PCA was documented as normal.

### Statistical analysis

SPSS Statistics Software (IBM SPSS Statistics for Windows, Version 27.0) was used to run the required analyses, and *p*-values < 0.05 were considered statistically significant. The relationship was analyzed for each artery. Correlation coefficients (*R*) were calculated using nonparametric correlation analysis (Spearman’s correlation) to assess the relationship between the readers’ interpretations and the DSA results. A weak correlation was defined as a correlation coefficient between 0.1 and 0.3, a moderate correlation between 0.3 and 0.7, a relatively high correlation between 0.7 and 0.9, and a very high correlation above 0.9. We performed receiver operating characteristic (ROC) analysis on the readers’ interpretations separately to evaluate whether the addition of CTP images improved diagnostic performance. An increase in the area under the curve (AUC) in the ROC curve analysis was considered to be indicative of improved diagnostic performance. A paired t-test was used to analyze the reading times of the two readers.

## Results

### The addition of CTP improved the diagnostic performance of CTA

The correlation coefficients for each intracranial artery before and after CTP are presented in Table 1. After the addition of CTP for both readers, the data indicated an improvement in diagnostic performance for ICA, MCA, ACA, VA, and PCA, but a negligible decrease for BA. The diagnostic performance was very good for the ICA, MCA, and BA (*R* > 0.7), and slightly worse, but good, for the ACA and VA (0.3 < *R <* 0.7). The data indicated an incorrect diagnosis for PCA (*R* < 0.1), whether reading CTA images alone (*R* = -0.103, *p* = 0.47 for both R1 and R2), or reading a combination of CTA images and CTP images (R1: *R* = 0.059, *p* = 0.68; R2: *R* = 0.069, *p* = 0.63). When R2 read the CTA images, the interpretation of the ACA had a weak correlation with the DSA data (*R* = 0.281, *p* = 0.07), which was not satisfactory. After the addition of CTP, the interpretation of the ACA had a moderate correlation with the DSA data (*R* = 0.689, *p* < 0.05), which was much better than before.

**Table 1:** Correlation coefficients (*R*) between readers’ interpretation and DSA results

Radiologist	Images	ICA *N* = 42	MCA *N* = 42	ACA *N* = 42	VA *N* = 52	BA *N* = 26	PCA *N* = 52
R1	CTA	0.665*	0.811*	0.455*	0.355*	0.799*	-0.103 (0.47)
	CTA + CTP	0.786*	0.812*	0.689*	0.398*	0.782*	0.059 (0.68)
R2	CTA	0.575*	0.802*	0.281	0.354*	0.832*	-0.103 (0.47)
	CTA + CTP	0.760*	0.908*	0.689*	0.501*	0.817*	0.069 (0.63)

DSA, digital subtraction angiography; ICA, internal carotid artery; MCA, middle cerebral artery; ACA, anterior cerebral artery; VA, vertebral artery; BA, basilar artery; PCA, posterior cerebral artery; CTA, computed tomographic angiography; CTP, computed tomographic perfusion

Values in parentheses are *p*-values. *p < 0.05 indicating the significance of the correlation. Correlation coefficient strength was characterized as follows: weak (0.1 ≤ *R* < 0.3), moderate (0.3 ≤ *R* < 0.7), relatively high (0.7 ≤ *R* < 0.9), and very high (*R* ≥ 0.9)

### The addition of CTP improved the diagnostic accuracy of CTA

**Fig. 2:**
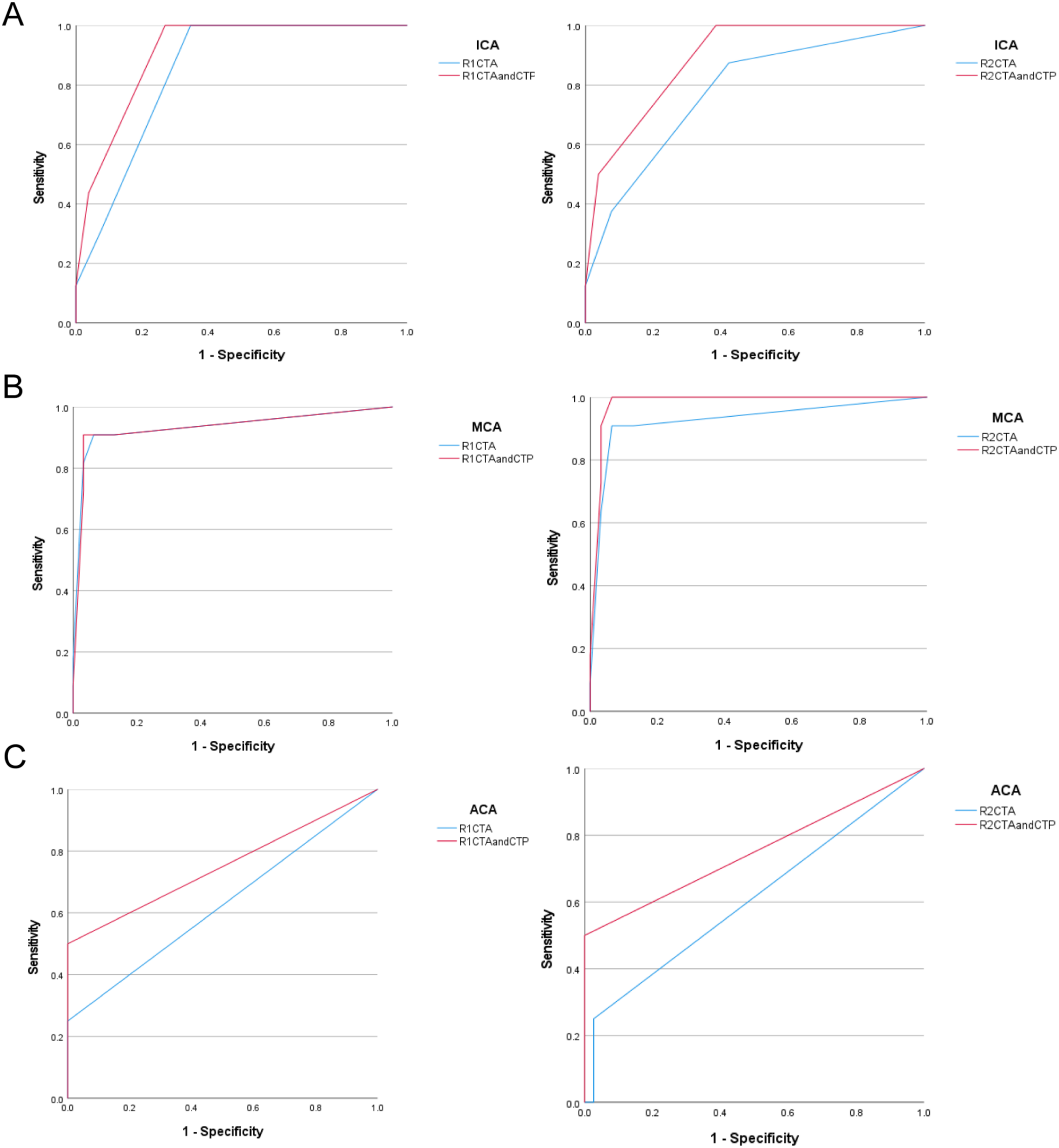
ROC curves evaluated the diagnostic value of before (CTA) and after (CTA and CTP) in patients’ ICA (**A**), MCA (**B**), and ACA (**C**) from two readers (R1 and R2). ICA, internal carotid artery; MCA, middle cerebral artery; ACA, anterior cerebral artery

**Fig. 3:**
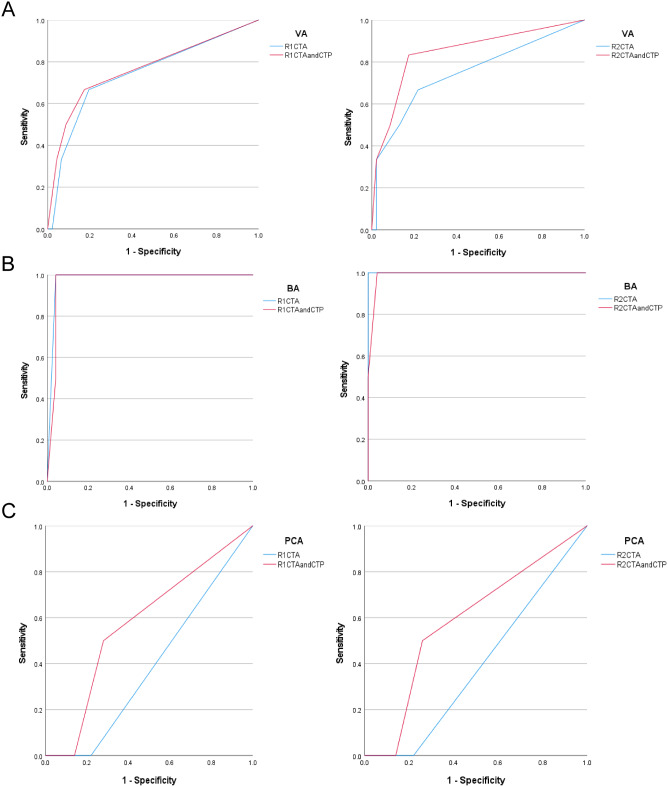
ROC curves evaluated the diagnostic value of before (CTA) and after (CTA and CTP) in patients’ VA (**A**), BA (**B**), and PCA (**C**) from two readers (R1 and R2). VA, vertebral artery; BA, basilar artery; PCA, posterior cerebral artery

Subsequently, ROC curve was used to evaluate the diagnostic value of intracranial arteries before and after the addition of CTP (CTA vs. CTA + CTP) (Figs. 2A-C and 3A-C). ROC analysis indicated excellent accuracy in analyzing the ICA, MCA, and BA at baseline and after the addition of CTP maps (*p* < 0.05). The AUC increased after the addition of CTP images for the ICA (R1 and R2), MCA (R2), ACA (R1 and R2), VA (R1 and R2), and PCA (R1 and R2), although the accuracy was poor for the ACA (*p* > 0.05) and PCA (*p* > 0.05). There was a slight decrease in the AUC for the MCA (R1) and BA (R1 and R2) after the addition of CTP images (Table 2).

**Table 2:** Area under the curve (AUC) of ROC analysis of each intracranial artery before and after addition of CTP

Radiologist	Images	ICA *N* = 42	MCA *N* = 42	ACA *N* = 42	VA *N* = 52	BA *N* = 26	PCA *N* = 52
R1	CTA	0.847*	0.934*	0.625 (0.42)	0.743 (0.06)	0.979*	0.390 (0.601)
	CTA + CTP	0.907*	0.933*	0.750 (0.10)	0.764*	0.969*	0.575 (0.721)
R2	CTA	0.776*	0.927*	0.609 (0.48)	0.748 (0.05)	1.000*	0.390 (0.601)
	CTA + CTP	0.887*	0.981*	0.750 (0.10)	0.846 *	0.990*	0.585 (0.686)

DSA, digital subtraction angiography; ICA, internal carotid artery; MCA, middle cerebral artery; ACA, anterior cerebral artery; VA, vertebral artery; BA, basilar artery; PCA, posterior cerebral artery; CTA, computed tomographic angiography; CTP, computed tomographic perfusion

Values in parentheses are p-values. *p < 0.05 indicating the significance of ROC curve

### The combination of CTP and CTA decreased the interpretation time of readers

**Fig. 4:**
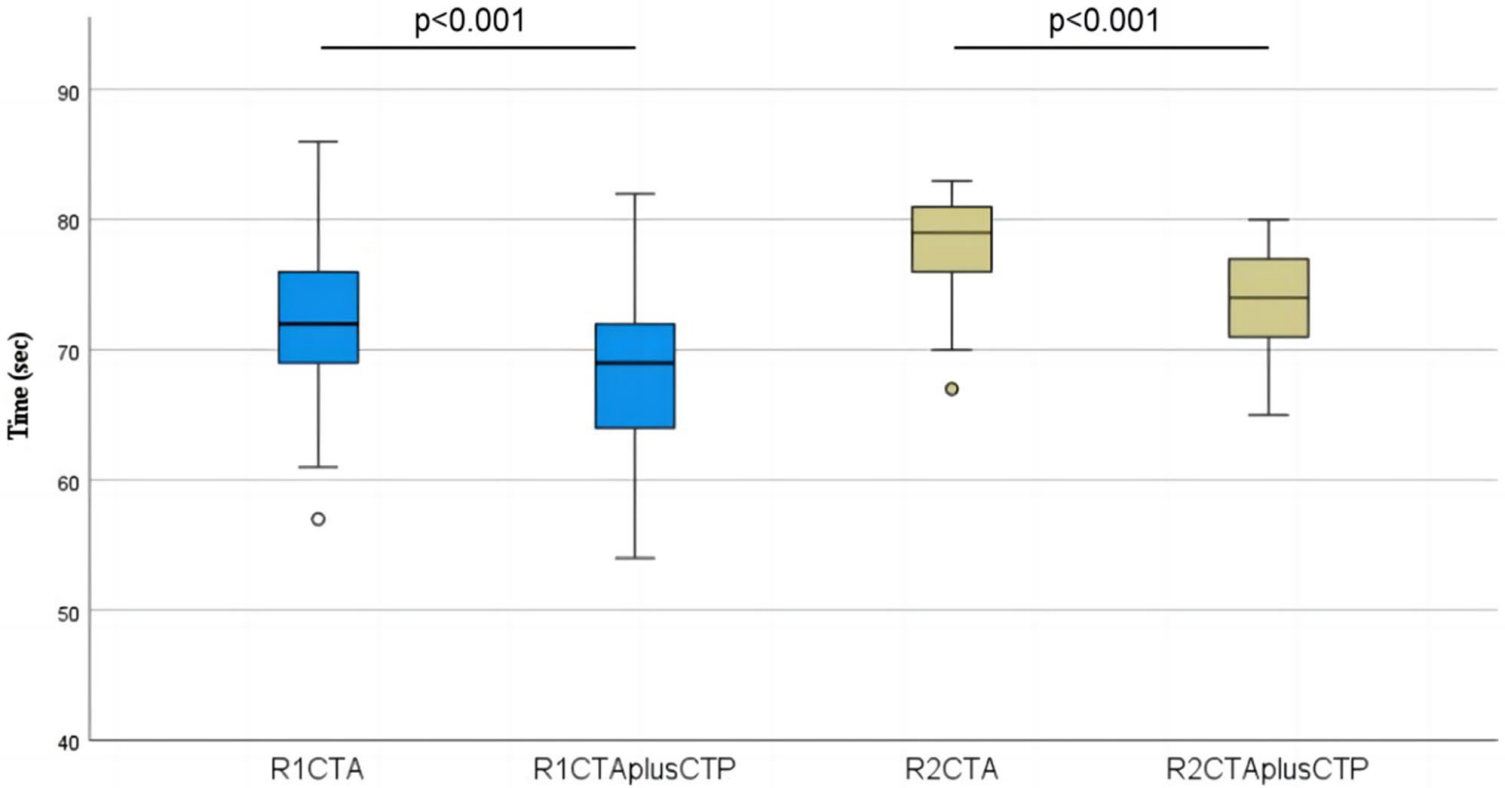
Box and whisker plots showed differences in interpretation times for the two readers before and after the addition of CTP images

We conducted a paired t-test to compare the reading times of the two readers and the result was shown in Fig. 4. The mean interpretation time decreased from 72.4 ± 6.1 s using CTA to 67.7 ± 6.4 s using the combination of CTA and CTP (*p* < 0.001) for R1, and from 77.7 ± 3.8 s to 72.6 ± 4.7 s for R2 (*p* < 0.001). The above results suggested the interpretation times for the two readers was significantly shortened after the addition of the CTP images.

## Discussion

Previous review revealed that CTA/CTP is a fast and convenient method for rapid triage of acute stroke patients, and its accuracy in diagnosing ischemia and locating thrombi proximal or distal to intracranial blood vessels far exceeds that of clinical examination, which is convenient for guiding patient treatment [9]. Tan et al. proposed that CTP combined with CTA could help accurately evaluate the occlusional site, infarct core, salvageable brain tissue and collateral circulation in acute stroke patients [10]. In our study, after adding CTP, the accuracy of diagnosing the ICA, ACA, VA, and PCA increased for both readers, which is consistent with the results of the two statistical analyses. The first nonparametric test indicated that the diagnostic ability for MCA was strengthened, whereas the second classification test suggested an improved diagnostic ability for R2 and a very slight decrease in diagnostic ability for R1, but still maintained a high level of performance. Considering that the classification test lost the grading data for DSA diagnosis, we adopted the first statistical result, which indicated that the diagnostic ability for the MCA improved after adding CTP. Surprisingly, both statistical analyses indicated a slight decrease in the accuracy of BA diagnosis after the addition of CTP. We speculate that a larger sample size may be required to correct for possible biases.

Both statistical methods indicated that the diagnostic performance of PCA was poor, regardless of whether it was performed before or after the addition of CTP. The reason for this consistently poor performance may be that the assessment of stenosis of the medium vasculature in such high-risk regions by DSA is not very accurate, as the posterior cranial fossa has a variety of structures with very different densities on CT images, and is prone to artefacts at the junction of two substances with widely differing densities. Despite its poor diagnostic ability, diagnostic performance improved after the addition of CTP. The first nonparametric test indicated that R2 had poor diagnostic ability for the ACA when interpreting CTA, but the diagnostic ability was significantly enhanced after adding CTP. Upon reviewing the raw data, we found that this result was due to the high variance in the ACA, and R2 was prone to misclassifying congenital absence as severe stenosis or occlusion when interpreting CTA alone. This positive bias significantly decreased after the addition of CTP data.

Previous studies on CTP have focused on the infarct region and volume or the responsible blood vessels in patients with acute ischemic stroke [5, 6, 11, 12]. No specific study has quantified the impact of additional CTP images on the diagnostic performance in non-stroke patients or regions. In clinical practice, we observed that CTP can provide more information to patients with asymptomatic intracranial arterial stenosis. If Tmax, a highly sensitive indicator, is prolonged while CBV and CBF are normal, this suggests possible severe vascular disease. If Tmax, CBV, and CBF are all normal, and CTA images show severe stenosis or occlusion, it suggests chronic vascular occlusion or Moyamoya disease. Our data show that additional CTP images can improve diagnostic performance (for ICA, MCA, ACA, VA, and PCA) and interpretation speed for identifying intracranial large and medium vessel stenosis or occlusion on CTA. This non-invasive diagnostic tool can provide a more reliable reference than CTA alone for physicians to develop individualized prevention strategies for stroke.

**Fig. 5:**
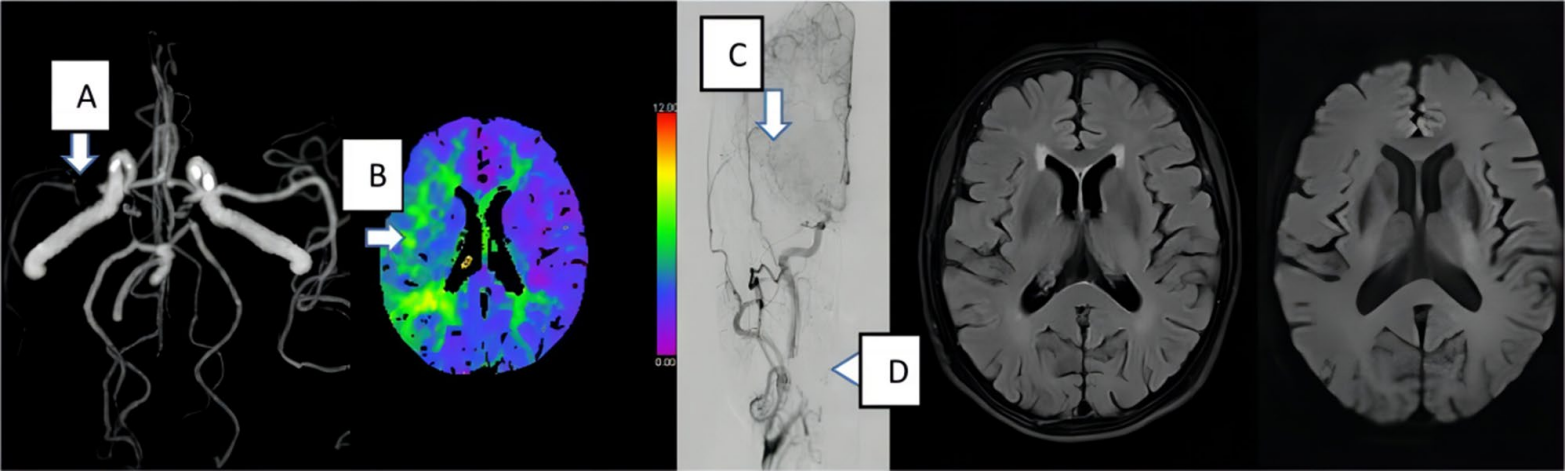
CTA images of a patient, suggesting severe stenosis of the right MCA (arrow **A**), and CTP images suggesting a large area of prolonged Tmax in the right MCA perfusion region (arrow **B**). The DSA image indicates occlusion of the right MCA (arrow **C**). The area indicated by triangle C was the location of the patient’s denture. There was no previous brain infarction apparent on the MRI T2FLAIR image, or acute ischemic stroke on the MRI DWI image. The interpretation times of the CTA images of the patient were 73 s for R1 and 81 s for R2. After the addition of CTP maps, the interpreting times were 71 s for R1 and 78 s for R2

Considering that incorporating CTP data leads to greater image content being interpreted by readers, it is surprising that the statistical results indicate a decrease in interpretation time. We speculate that CTP provides perfusion information, particularly with highly sensitive Tmax, which increases the confidence of readers in assessing the extent of cerebral vascular lesions, thus enabling faster interpretation (Fig. 5). In the second round of interpretation (CTA and CTP), R1 adjusted their reading habits from CTA first, then CTP, to CTP first, and then CTA, after interpreting seven samples. R1 stated that adjusting the order in this manner made it easier, similar to knowing the answer, before attempting to answer the question. R2 stated that normal perfusion images in the second round of interpretation provided greater certainty regarding normal blood vessels and reduced concerns regarding missing significant local lesions. The time saved during the reading process can offset the additional examination time required for performing CTP scans compared to performing CTA scans alone. This allows physicians to reduce the delay in making treatment decisions caused by the advanced imaging examinations.

Our study used a low-radiation-dose CTP: CT dose index of 166 mGy [13]. Lower radiation doses result in less radiation damage to patients. CTA images can be a part of the CTP data, and can improve the diagnostic performance without increasing the radiation dose or contrast agent injection using existing CTP datasets. Our CTP scans were performed at different frame rates; the first 19 sets were 1.5 s, and the remaining six sets were 3 s. This parameter setting allowed us to obtain high-quality images while minimizing the radiation dose to the patients. Additionally, by using a frame rate of 1.5 s for the initial set of images, it is convenient to remove individual motion artifact images during CTP examination. The remaining images remained within a time gap of 3 s, meeting the frame rate requirements outlined in the CTP operating guidelines [14]. We did not find any CTP data with motion artifacts during patient selection. This study also provides supporting evidence for the feasibility and accuracy of low-radiation-dose CTP imaging for the detection of intracranial large/medium vessels (ICA, MCA, and BA).

The limitations of the current study include its retrospective nature and the relatively small sample size. Inevitably, there is some bias in this study, rearranging the sample after one week may not eliminate the recall bias and the readers’ skill improvement during this period may contribute possible bias, but the bias is slight as the readers’ skills are mature and stable. Additionally, the absence of vasculature subdivision is an important limitation. Future work should further expand the sample size to perform vasculature subdivision, which contributes to personalize diagnostic work-up and therapies in asymptomatic intracranial arterial stenosis.

## Conclusion

Additional CTP images improved the accuracy of interpreting CTA images (ICA, MCA, ACA, VA, and PCA) and reduced the interpretation time in asymptomatic intracranial arterial stenosis. CTP images obtained using low-dose radiation, along with the derived CTA images, showed good diagnostic accuracy for intracranial large-vessel stenosis or occlusion. These findings support the use of CTP imaging in patients with asymptomatic intracranial arterial stenosis. Further large sample prospective study is needed to confirm the diagnostic value of CTP images in vasculature subdivision of symptomatic intracranial arterial stenosis.

## Data Availability

The datasets used and/or analysed during the current study available from the corresponding author on reasonable request.
